# Rack1 mediates Src binding to drug transporter P-glycoprotein and modulates its activity through regulating Caveolin-1 phosphorylation in breast cancer cells

**DOI:** 10.1038/s41419-019-1633-y

**Published:** 2019-05-21

**Authors:** Yanling Fan, Weiyao Si, Wei Ji, Zhiyong Wang, Zicong Gao, Ran Tian, Weijie Song, He Zhang, Ruifang Niu, Fei Zhang

**Affiliations:** 1 Public Laboratory, Tianjin Medical University Cancer Institute and Hospital, National Clinical Research Center for Cancer, Tianjin, 300060 China; 2 Key Laboratory of Cancer Prevention and Therapy, Tianjin, 300060 China; 3 Tianjin’s Clinical Research Center for Cancer, Tianjin, 300060 China; 4 Key Laboratory of Breast Cancer Prevention and Therapy, Tianjin Medical University, Ministry of Education, Tianjin, 300060 China

**Keywords:** Breast cancer, Cancer therapeutic resistance

## Abstract

The failure of chemotherapy and the emergence of multidrug resistance (MDR) are the major obstacles for effective therapy in locally advanced and metastatic breast cancer. Overexpression of the drug transporter P-glycoprotein (P-gp) in cancer cells is one of the main causes of MDR due to its ability to efflux anticancer drugs out of cells. Although the signaling node that regulates the expression of P-gp has been intensively investigated; the regulatory mechanism underlying P-gp transport activity remains obscure. Herein, we reported that Rack1 and tyrosine kinase Src confer drug resistance through modulating the transport function of P-gp without altering its protein level. We provide evidences that Rack1 and Src regulate P-gp activity by modulating caveolin-1 (Cav1) phosphorylation. Importantly, Rack1 acts as a signaling hub and mediates Src binding to P-gp, thereby facilitating the phosphorylation of Cav1 by Src and abolishing the inhibitory effect of Cav1 on P-gp. Taken together, our results demonstrate the pivotal roles of Rack1 and Src in modulating P-gp activity in drug-resistant cells. Our findings also provide novel insights into the mechanism regulating P-gp transport activity. Rack1 may represent a new target for the development of effective therapies for reversing drug resistance.

## Introduction

Breast cancer is the most frequent malignant disease among females^1,2^. Chemotherapy is a major treatment strategy for patients with locally advanced or metastatic breast cancer. Nevertheless, a large number of patients gradually lose response to chemotherapeutic agents. More formidably, cancer cells become resistant to a variety of anticancer drugs and even to those they have never encountered, a phenomenon defined as multidrug resistance (MDR)^3^. Although great effort has been made in improving the therapeutic strategies for drug-resistant cancers, breast cancer remains the leading cause of cancer death among women. Therefore, the underlying mechanisms that enable cancer cells to acquire resistance to anticancer drugs must be identified and novel methods to reverse drug resistance in cancer must be developed.

The best characterized mechanism of MDR is the overexpression of ATP-binding cassette (ABC) transporters in cancer cells^4,5^, including P-glycoprotein (P-gp), breast cancer resistance protein (BCRP), and MDR-associated protein 1 (MRP1), which function as drug pumps that efflux drugs out of cells^3,6,7^. Among these transporters, P-gp is the first identified and the most well-investigated protein. The increased expression of P-gp in tumor tissues is frequently associated with poor response to chemotherapy in many kinds of carcinomas^8,9^. Therefore, identifying the underlying pathways through which cancer cells upregulate P-gp expression and depicting the detailed mechanisms for regulating P-gp activity will provide more effective therapeutic strategies. Intensive studies have demonstrated that several key cellular signaling and critical transcription factors are involved in the upregulation of ABCB1/P-gp^10,11^; however, the regulatory mechanism of P-gp transport function is poorly studied.

The functional activity of P-gp is regulated by protein–protein interactions. A well-known protein that can modulate P-gp activity is caveolin-1 (Cav1)^12,13^. Although elevated Cav1 expression has been observed in several MDR cancer cells, high level of Cav1 does not confer MDR^14–16^. Conversely, Cav1 interacts with P-gp and suppresses its transport function^17–19^. Knockdown of Cav1 or attenuation of P-gp/Cav1 interaction enhances the transport activity of P-gp. The binding capacity of Cav1 to P-gp is modulated by tyrosine phosphorylation^19^ and Cav1 phosphorylation is regulated by the Src tyrosine kinase^20^. Elevated Src activity has been observed in many types of cancer and the blockage of Src activity re-sensitizes cancer cells to anticancer drugs^21–24^. However, the detailed mechanism whereby Src phosphorylates Cav1 and contributes to drug resistance remains poorly understood.

Receptor for activated C kinase 1 (Rack1) is a multifunctional scaffold protein involved in various pivotal cellular processes^25–30^. The aberrant expression of Rack1, which acts as a tumor promoter or suppressor in a tissue-type and context-dependent manner, has been observed in many types of carcinomas. Rack1 exerts different effects on cancer progression and might depend on its binding partners^28^. Rack1 has been reported to interact with multiple cellular receptors, including IGF-1R, FGFR1, androgen receptor, and interferon receptor^31–34^. In addition, Rack1 also interacts with various protein kinases, including PKC, PKM2, FAK, MEK, and MKK7^35–37^. One of the most studied binding partners of Rack1 is Src^26,37,38^. We previously showed that Rack1 is a novel binding protein of P-gp and Rack1 mediates Src binding to P-gp and promotes invasiveness in drug-resistant cells^39^. However, whether this interaction is involved in drug resistance remains unknown. In this study, we further characterized the interaction among P-gp, Rack1, Src, and Cav1 in drug-resistant cells and investigated whether this protein complex is implicated in drug resistance. We showed that Rack1 and Src positively regulate drug resistance by modulating P-gp activity without altering its protein level. Furthermore, Rack1 and Src are required for Cav1 phosphorylation and Rack1 mediates Src binding to P-gp, thereby facilitating the phosphorylation of Cav1 by Src. Therefore, Rack1 is a critical regulator in drug resistance.

## Results

### Knockdown of Rack1 enhances drug sensitivity in MDR cells

**Fig. 1:**
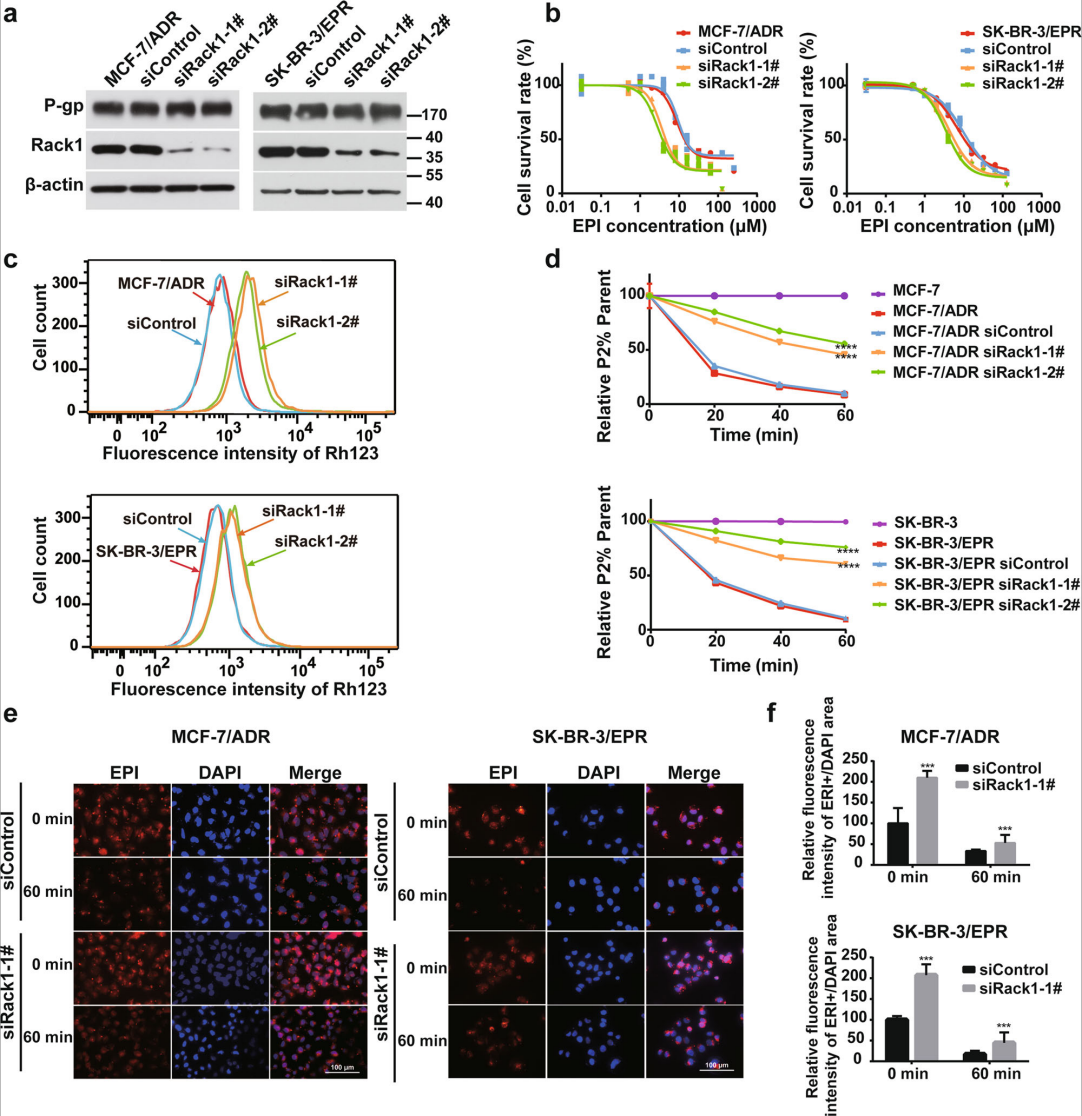
Rack1 knockdown enhances drug sensitivity in MDR cells. **a** Western blotting analysis of P-gp expression in MCF-7/ADR and SK-BR-3/EPR cells transfected with negative control or Rack1-specific siRNAs; β-actin was used as a loading control. **b** Knockdown of Rack1 in two MDR cells enhanced the sensitivity to EPI in comparison with control cells. Cells were treated with different concentrations of EPI and the cell viability was determined by a CCK8-based assay. The assay was performed in triplicates for each EPI concentration and repeated thrice. IC_50_ was calculated by using the GraphPad Prims 6.0 software. **c** Knockdown of Rack1 in two drug-resistant cells increases cellular Rh123 retention in comparison with wild-type and control cells as measured by flow cytometry. **d** Knockdown of Rack1 in two drug-resistant cells decreases the efflux rate of Rh123 compared with control cells. Cells were incubated in Rh123 dye-containing medium for 30 min, then washed with PBS, and incubated in Rh123-free medium for 0, 15, 30, 45, and 60 min. At each time point, cells were immediately detected by using flow cytometry. The assays were carried out in triplicate and repeated three times. Data were shown as mean ± SD, ****P < 0.0001 vs. siControl in MDR cells, statistical analysis was performed by two-way ANOVA. **e** Knockdown of Rack1 in MDR cells increased cellular EPI retention compared with control cells. Cells were initially incubated with 2 μM of EPI for 2 h and then incubated with EPI-free medium for additional 1 h. Afterward, the cells were counterstained with 1.0 ng/mL of DAPI for nuclei. Images were captured by fluorescence microscope. **f** Quantification of EPI fluorescence intensity in Fig. 1e by using Image J (NIH, Bethesda, MD, USA) software. ***P < 0.001 vs. siControl, data are presented as mean ± SD, statistical analysis was calculated by two-way ANOVA

To determine whether Rack1 contributes to drug susceptibility, we used small interfering RNAs (siRNAs) to downregulate the expression of Rack1 in MDR breast cancer cells (Fig. 1a). Then, cell counting kit-8 (CCK8)-based assay was used to examine the sensitivity to epirubicin (EPI) in Rack1-silenced cells. Figure 1b showed that Rack1 knockdown significantly decreased the IC_50_ value compared with that of the control group (Supplementary Tables 1 and 2). Interestingly, the silence of Rack1 had no significant effect on P-gp expression compared with that of the control group (Fig. 1a). We supposed that Rack1 knockdown may attenuate P-gp activity in breast cancer cells. To test this possibility, we used Rh123 efflux assay to evaluate the function of P-gp after Rack1 reduction. As shown in Fig. 1c, Rack1 knockdown markedly enhanced the retention of intracellular Rh123 compared with that of the control cells. Moreover, ~50% of Rh123 dye remained accumulated in Rack1-silenced cells after 60 min of incubation in the dye-free medium, whereas >50% of the dye was removed from the control cells in 20 min and only <10% was retained in the control cells after the 60 min efflux (Fig. 1d). Hence, the increased drug sensitivity after Rack1 knockdown was due to the decrease in P-gp activity. Furthermore, we performed fluorescent microscopy to detect the intrinsically fluorescence of the P-gp substrate EPI. As shown in Fig. 1e, f, the incubation of 2 μM of EPI for 2 h led to the apparently intracellular accumulation of EPI in Rack1-silenced cells. When the cells were further incubated in a drug-free medium for additional 1 h, drug was nearly completely removed from the control cells; however, significant cellular drug retention can also be observed in the Rack1-silenced cells, indicating the inhibition of P-gp activity. Collectively, these data demonstrate that Rack1 knockdown enhances drug sensitivity by suppressing P-gp activity without affecting its protein level.

### Knockdown of Src increases chemosensitivity in MDR cells

**Fig. 2:**
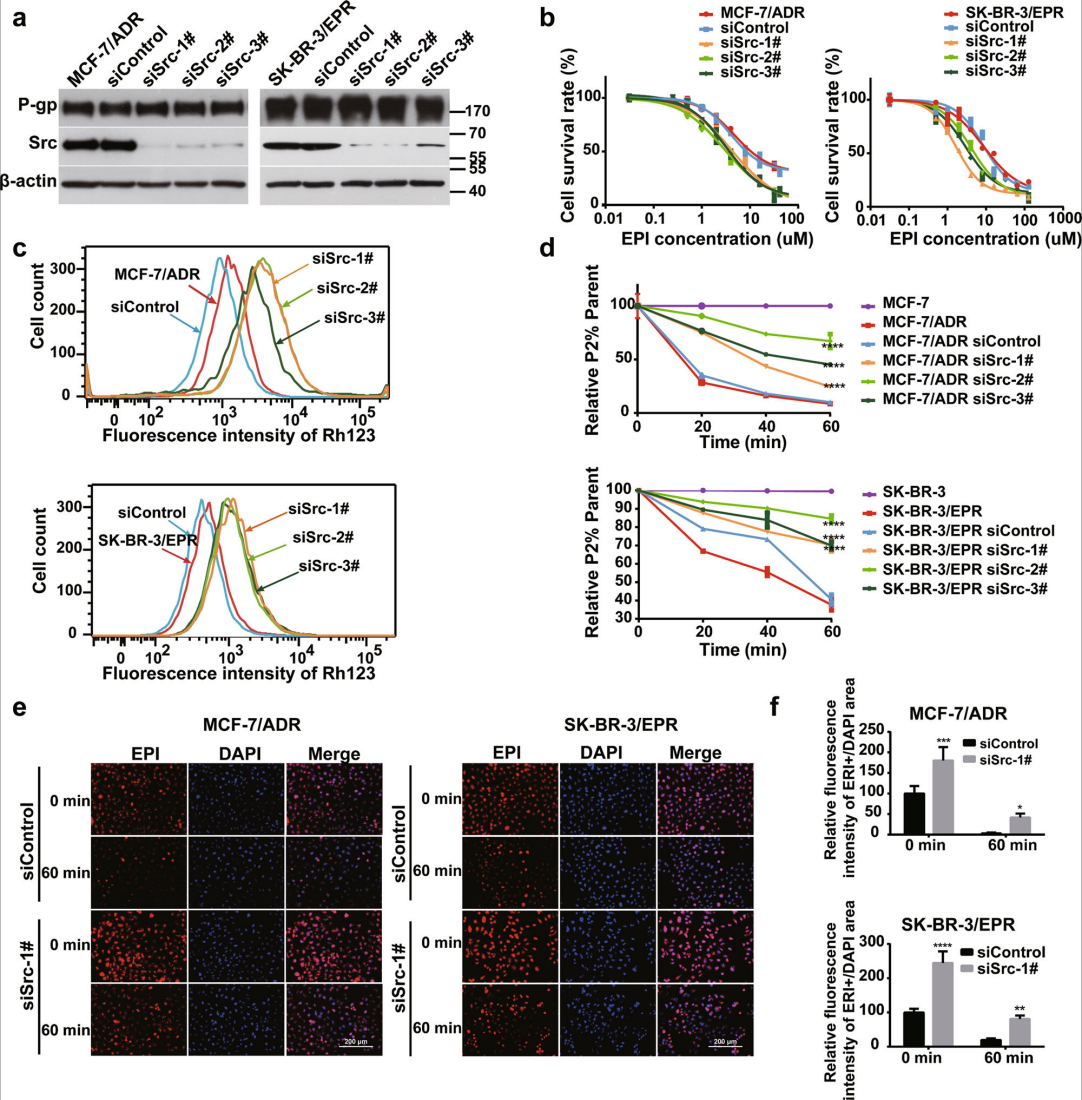
Src knockdown increases drug sensitivity in MDR cells. **a** Western blotting analysis of P-gp expression in two drug-resistant cells transfected with negative control or three Src-specific siRNAs; β-actin was used as a loading control. **b** Knockdown of Src in two drug-resistant cells significantly enhanced the sensitivity to EPI in comparison with control and wild-type cells. Drug sensitivity assay was determined using a CCK8-based assay. **c** Knockdown of Src in two drug-resistant cells increased cellular Rh123 retention in comparison with wild-type and control cells as measured by flow cytometry. **d** Knockdown of Src in two drug-resistant cells decreases the efflux rate of Rh123 compared with control cells. Data are presented as mean ± SD, ****P < 0.0001. **e** Knockdown of Src in drug-resistant cells increased cellular EPI retention compared with control cells. **f** Quantitative analysis of fluorescence intensity of EPI in Fig. 2e, *P < 0.05, **P < 0.01, ***P < 0.001, ****P < 0.0001 vs. control, data were analysed via two-way ANOVA

Src is a P-gp-binding protein^39^; it also interacts with Rack1 in many cell types^26,38,40^. To investigate whether Src is associated with P-gp activity and drug resistance in breast cancer cells, we silenced the expression of Src by using siRNAs (Fig. 2a). As shown in Fig. 2b, Src knockdown decreased the survival rate of resistant cells exposed to EPI. Considering that P-gp expression level remained unchanged after Src knockdown (Fig. 2a), we determined the efflux pump function in Src-silenced cells by flow cytometry. As shown in Fig. 2c, the accumulation of intracellular Rh123 dye significantly increased in Src knockdown cells than in the control cells, indicating a reduction of P-gp activity. Consistently, Rh123 dye efflux time kinetics analysis also showed that Src-silenced cells had a lower capacity to exclude Rh123 dye compared with that of control cells (Fig. 2d). Moreover, knockdown of Src significantly reduced the efflux of EPI out of cells, resulting in a marked intracellular accumulation of EPI (Fig. 2e, f). Overall, these results demonstrated that Src regulates drug resistance in breast cancer through modulating the P-gp activity.

**Fig. 3:**
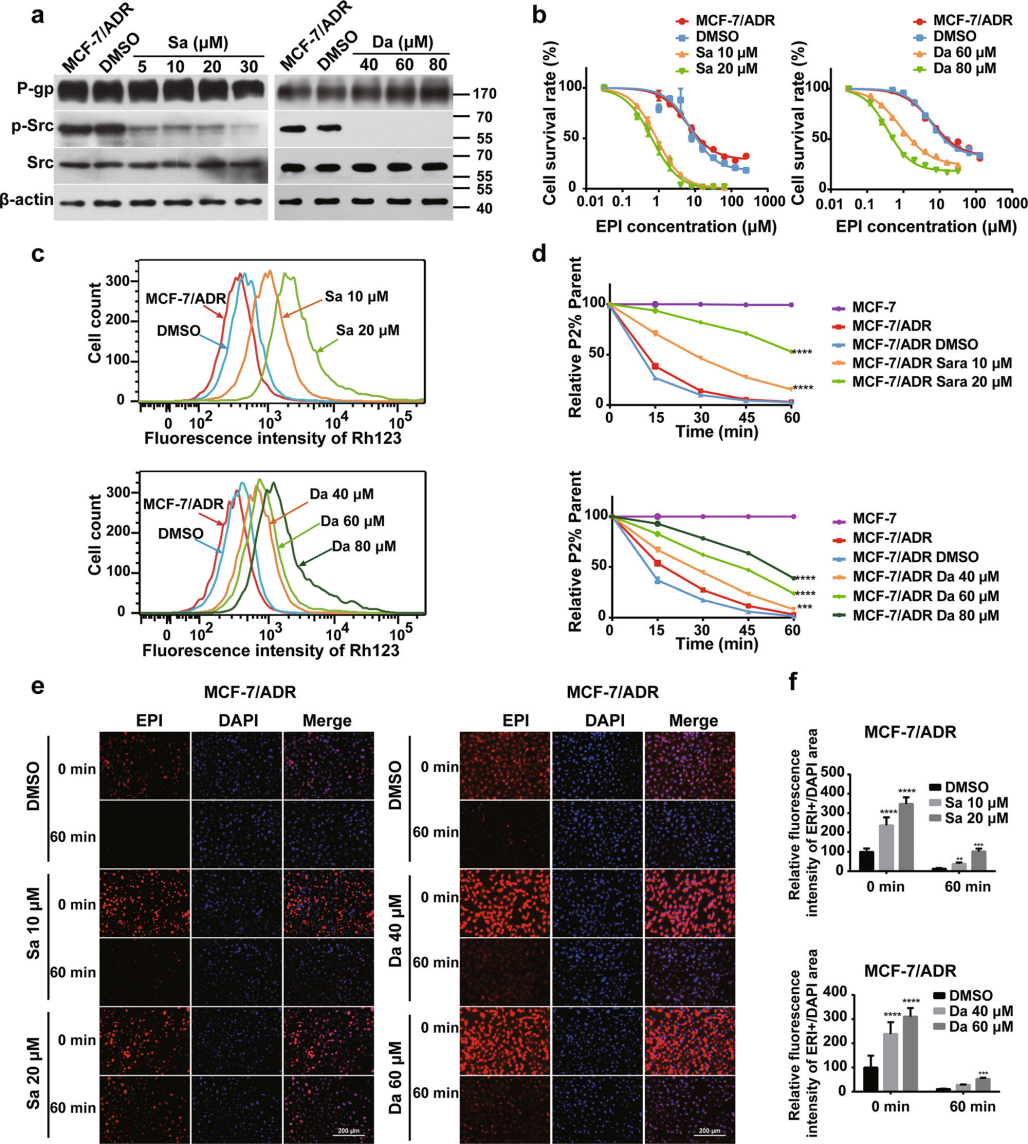
Inhibition of Src kinase activity increases chemosensitivity in MDR cells. **a** Western blotting analysis of the expression of P-gp, total Src, and phosphorylated Src in drug-resistant cancer cells after Saracatinib or Dasatinib treatment for 24 h. **b** Treatment with Src inhibitors in MDR cells significantly enhanced drug sensitivity to EPI in comparison with control cells. The cells were pretreated with Src inhibitors for 24 h, then different concentrations of EPI were added into cells, and drug sensitivity assay was performed by CCK8-based assay as described above. Values were expressed as mean ± SD from three independent experiment. **c** Inhibition of Src activity by two inhibitors in resistant cells enhanced intracellular Rh123 retention in comparison with wild-type and control cells as measured by flow cytometry. **d** Src inhibitors treatment significantly decreases the efflux rate of Rh123 compared with control cells. Data were presented as mean ± SD, statistical analysis was performed using two-way ANOVA, ***P < 0.001, ****P < 0.0001 vs. solvent control. **e** Src inhibitors treatment significantly increased cellular EPI retention compared with control cells. **f** Quantification of EPI fluorescence intensity in Fig. 3e by using Image J (NIH, Bethesda, MD, USA) software. ***P < 0.001 vs. DMSO control, data are presented as mean ± SD, statistical analysis was calculated by two-way ANOVA

Furthermore, we treated MDR cancer cells with Src inhibitors to investigate whether Src activity is essential for resistance of cancer cells. As shown in Fig. 3a and Supplementary Fig. 1a, the two inhibitors significantly suppressed Src phosphorylation, indicating that the kinase activity is blocked. Src inhibition has no significant effect on P-gp expression, but the resistance to EPI was markedly decreased in inhibitor-treated cells relative to that in the control cells (Fig. 3b, Supplementary Fig. 1b, Supplementary Tables 3 and 4). Rh123 efflux assay showed that the proportion of high Rh123 cells significantly increased in the presence of Src inhibitors (Fig. 3c and Supplementary Fig. 1c). Moreover, Src inhibitors impaired the efflux capacity of resistant cells in a dose-dependent manner (Fig. 3d and Supplementary Fig. 1d). Thus, Src inhibitors efficiently prevented Rh123 efflux from resistant cells via inhibiting P-gp activity, resulting in its cellular retention. In addition, fluorescent microscopy showed that the intracellular accumulation of EPI was markedly elevated in the inhibitor-treated group compared with that in the control group (Fig. 3e, f and Supplementary Fig. 1e, f). Collectively, the Src kinase activity is necessary to the function of P-gp in resistant cells.

### Rack1 mediates the interaction between P-gp and Src

**Fig. 4:**
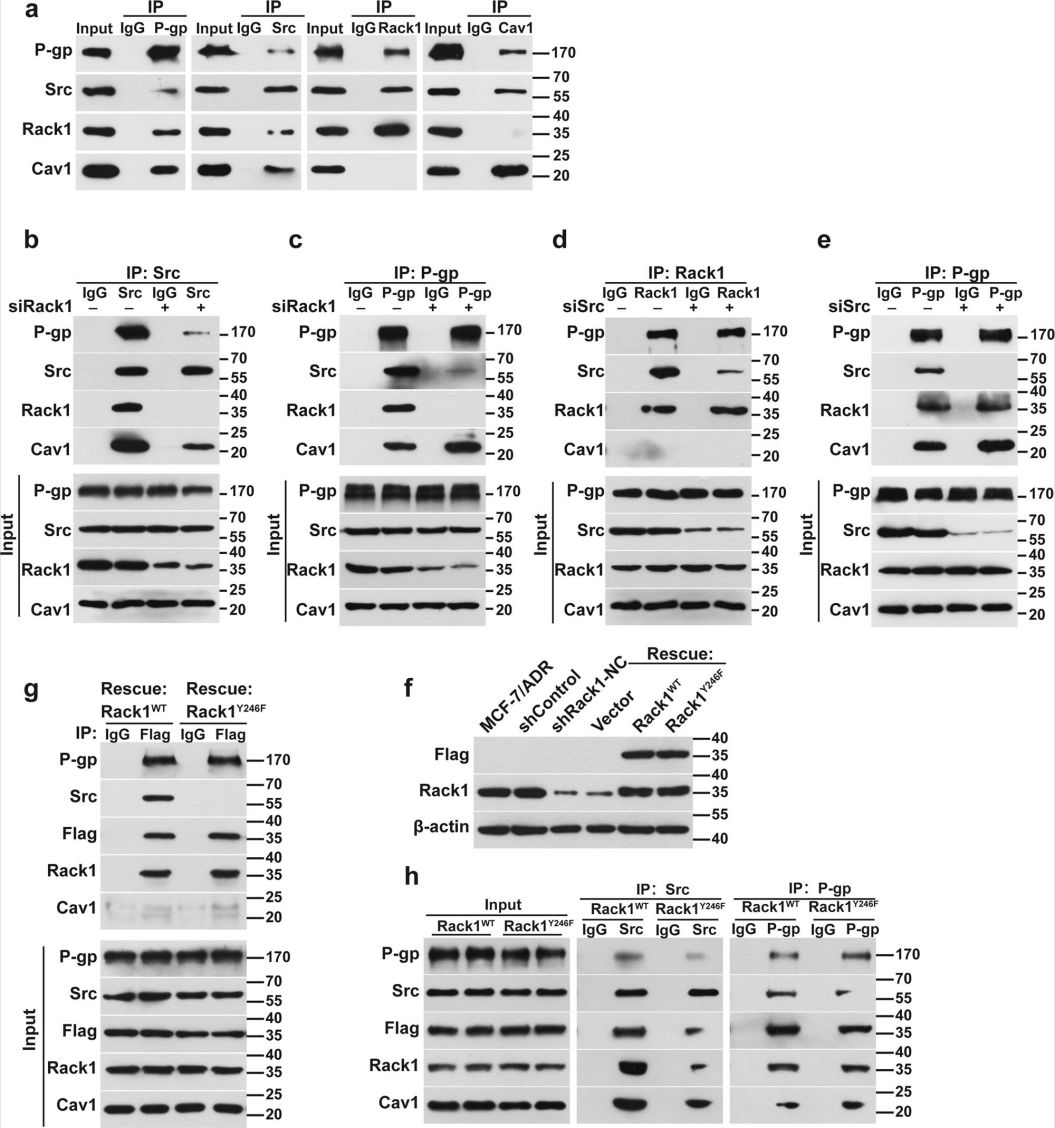
Rack1 mediates the interaction between P-gp and Src in drug-resistant cells. **a** Co-immunoprecipitation assay showed that endogenous P-gp and Src interacted with endogenous Rack1 and Cav1 in drug-resistant breast cancer cells, whereas Rack1 or Cav1 interacted with P-gp and Src, no interaction was detected between Rack1 and Cav1. MCF-7/ADR cells were lysed, immunoprecipitated with anti-P-gp, Src, Rack1, or Cav1 antibodies, and then analyzed by western blotting. **b** Rack1 knockdown decreased the interaction of Src with P-gp and Cav1 in MCF-7/ADR cells. Control and Rack1-silenced cells were lysed, immunoprecipitated with anti-Src antibody, and then analyzed by western blotting. **c** Silence of Rack1 expression attenuated ability of P-gp binding to Src, but enhanced the interaction between P-gp and Cav1. Control and Rack1-silenced cells were lysed, immunoprecipitated with anti-P-gp antibody, and then analyzed by western blotting. **d** Src knockdown has no significant effect on the binding of Rack1 to P-gp. Control and Src-silenced cells were lysed, immunoprecipitated with anti-Rack1 antibody, and then analyzed by western blotting. **e** Knockdown of Src has no significant effect on the interaction between P-gp and Rack1, whereas enhances the binding ability of Cav1 to P-gp. Control and Src-silenced cells were lysed, immunoprecipitated with anti-P-gp antibody, and then analyzed by western blotting. **f** Expression of Rack1 or its mutant Rack1^Y246F^ was effectively rescued in Rack1-depleted MCF-7/ADR cells. Rack1 expression was stably silenced by using an shRNA specifically targeting its noncoding region. Then Rack1-silenced cells were infected with lentivirus expressing Flag-tagged Rack1^WT^ and Rack1^Y246F^ mutant, and stable rescued cell lines were selected by using 50 μg/mL of hygromycin B. The cells were lysed and immunoblotted with anti-Flag and Rack1 antibodies. **g** Re-expression of Rack1^WT^, but not Rack1^Y246F^, can rescue the binding of Src to Rack1. Rack1^WT^- and Rack1^Y246F^-rescued cells were lysed, immunoprecipitated with anti-Flag antibody, and then analyzed by western blotting. **h** Re-expression of Rack1^Y246F^ decreased the interaction between P-gp and Src, but had no effect on its binding to P-gp. Rack1^WT^- and Rack1^Y246F^-rescued cells were lysed, immunoprecipitated with anti-Src or P-gp antibodies, and then analyzed by western blotting

To validate whether Rack1 mediates the interaction between Src and P-gp, we investigated the binding ability of Src to P-gp in Rack1-silenced cells through a co-immunoprecipitation (Co-IP) assay using anti-P-gp-, Src-, and Rack1-specific antibodies. As shown in Fig. 4a, b, anti-Src antibodies co-precipitated P-gp and Rack1 in cell lysates from control cells, whereas the interaction of Src with P-gp and Cav1 was notably attenuated in the Rack1-silenced cells. Consistently, a reciprocal Co-IP experiment using anti-P-gp antibodies also confirmed that the binding of P-gp with Src in Rack1-silenced cells is considerably hindered compared with that in the control cells (Fig. 4c). Interestingly, Rack1 silencing enhanced the interaction between P-gp and Cav1 (Fig. 4c). To further explore whether Src is required for the binding of Rack1 to P-gp, we performed Co-IP assay in Src-silenced cells. Interestingly, Src knockdown did not affect the interaction of P-gp with Rack1 (Fig. 4d, e), indicating that the interaction of Rack1 with P-gp was not affected in the Src-depleted cells. However, Src silencing enhanced the binding of P-gp to Cav1 (Fig. 4e). Given that Tyr246 is required for Rack1 interaction with Src^40^, we determined whether Tyr246 of Rack1 is necessary for the binding of Src to P-gp. We constructed two lentiviruses expressing either a Flag-tagged wild-type Rack1 (Rack1^WT^) or a Src binding-deficient Rack1 mutant (Rack1^Y246F^), and introduced these viruses into Rack1-silenced cells where Rack1 expression was stably silenced by an short hairpin RNA (shRNA) targeting its noncoding region. Figure 4f, g showed that the re-expression of Rack1^WT^ rather than Rack1^Y246F^ can rescue the binding of Src to Rack1. Rack1^Y246F^ can disrupt the interaction between P-gp and Src but has no effect on the interaction between P-gp and Rack1 (Fig. 4f–h). Collectively, Rack1 functions as a scaffold protein and mediates the interaction between P-gp and Src.

### Cav1 is phosphorylated by Src in Rack1-dependent manner

**Fig. 5:**
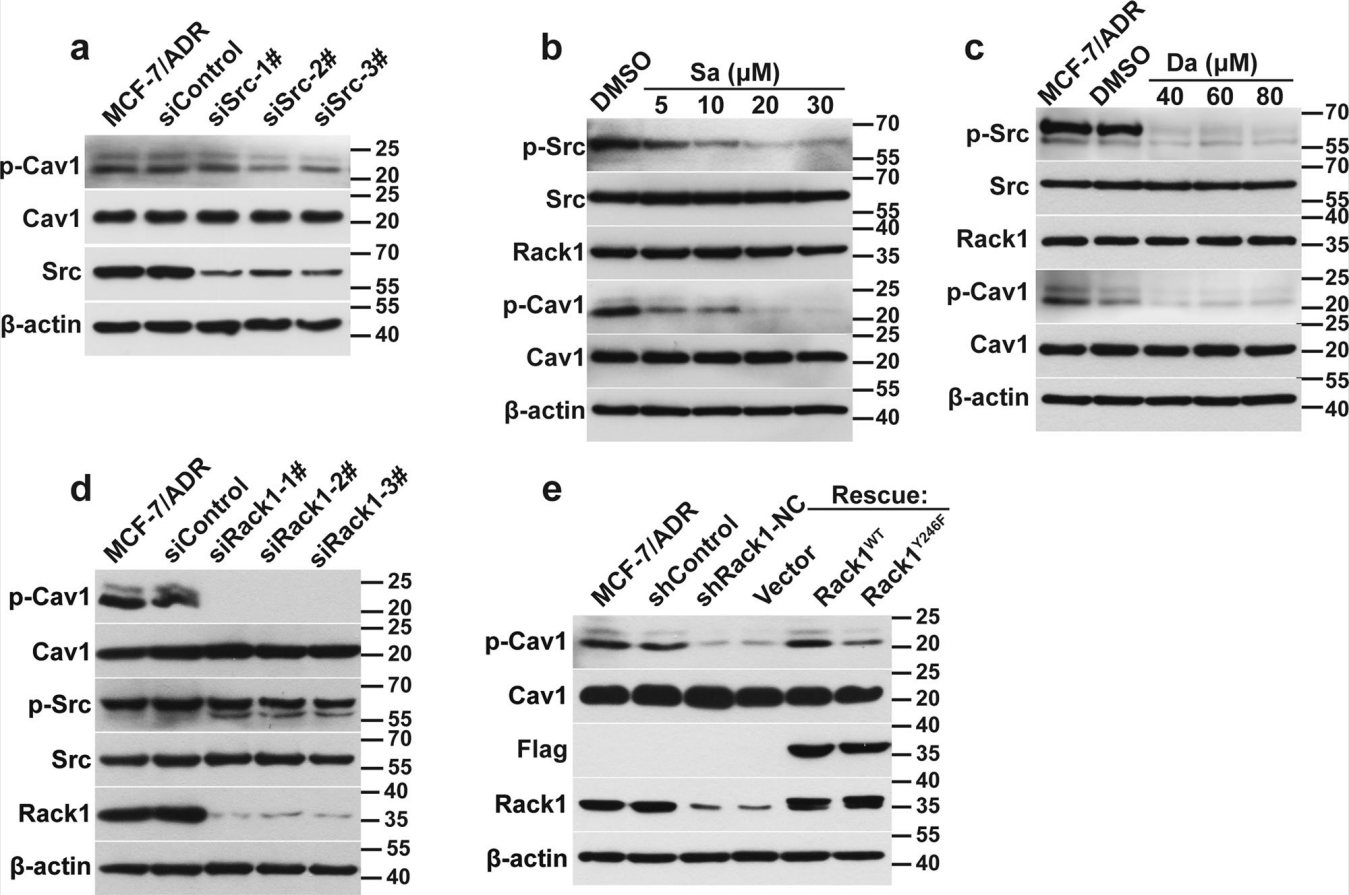
Cav1 is phosphorylated by Src in Rack1-dependent manner. **a** Src knockdown inhibited Cav1 tyrosine phosphorylation in MCF-7/ADR cells. Western blotting analysis of the expression level of Src, and total and phosphorylated Cav1 in cell lysates from drug-resistant cells transfected with negative control or three Src-specific siRNAs; β-actin was used as a loading control. **b** The blockage of Src activity with Saracatinib apparently inhibited Cav1 phosphorylation. Western blotting analysis of the expression level of Rack1, total and phosphorylated Src, and total and phosphorylated Cav1 in cell lysates from drug-resistant cells pretreated with Saracatinib for 24 h. **c** The blockage of Src activity with Dasatinib apparently inhibited Cav1 phosphorylation. Western blotting analysis of the expression level of Rack1, total and phosphorylated Src, and total and phosphorylated Cav1 in cell lysates from drug-resistant cells pretreated with Dasatinib for 24 h. **d** Rack1 knockdown inhibited Cav1 phosphorylation in MCF-7/ADR cells. Western blotting analysis of the expression level of Rack1, total and phosphorylated Src, and total and phosphorylated Cav1 in cell lysates from drug-resistant cells transfected with negative control or three Src-specific siRNAs; β-actin was used as a loading control. **e** The rescued expression of Rack1^WT^, but not Rack1^Y246F^ mutant, recovered Cav1 phosphorylation in Rack1-silenced cells. The Rack1 stable silenced cells were infected with lentivirus expressing Flag-tagged Rack1^WT^ or Rack1^Y246F^ mutant. Then wild-type, control, and Rack1^WT^- and Rack1^Y246F^-rescued cells were lysed and immunoblotted with anti-Flag, Rack1, Cav1, and phosphorylated Cav1 antibodies

To examine whether Src regulates the phosphorylation of Cav1, we silenced Src expression by using siRNAs. Src downregulation significantly suppressed the phosphorylation of Cav1 without affecting its total protein level (Fig. 5a). In addition, the Src inhibitors were also used to treat resistant cells. Figure 5b, c showed that the blockage of Src activity apparently inhibited Cav1 phosphorylation. Given that Rack1 is a binding partner of Src, to test whether Rack1 was associated with Cav1 phosphorylation, we silenced Rack1 expression by using siRNAs. Figure 5d showed that Rack1 knockdown inhibited the phosphorylation of Cav1. Interestingly, the phosphorylation of Src in the Rack1-silenced cells remained unchanged. To further determine whether Rack1 is required for Cav1 phosphorylation, we re-introduced the Flag-tagged Rack1^WT^ or Rack1^Y246F^ mutant lentivirus into the Rack1-silenced cells. As shown in Fig. 5e, the rescued expression of Rack1^WT^ recovered Cav1 phosphorylation nearly to the similar level as that observed in the control cells, whereas the re-expression of Rack1^Y246F^ mutant failed to rescue Cav1 phosphorylation. Taken together, these data suggested that the phosphorylation of Cav1 was regulated by Src in a Rack1-dependent manner in drug-resistant cells.

### Phosphorylation of Cav1 decreases its binding to P-gp and enhances P-gp activity

**Fig. 6:**
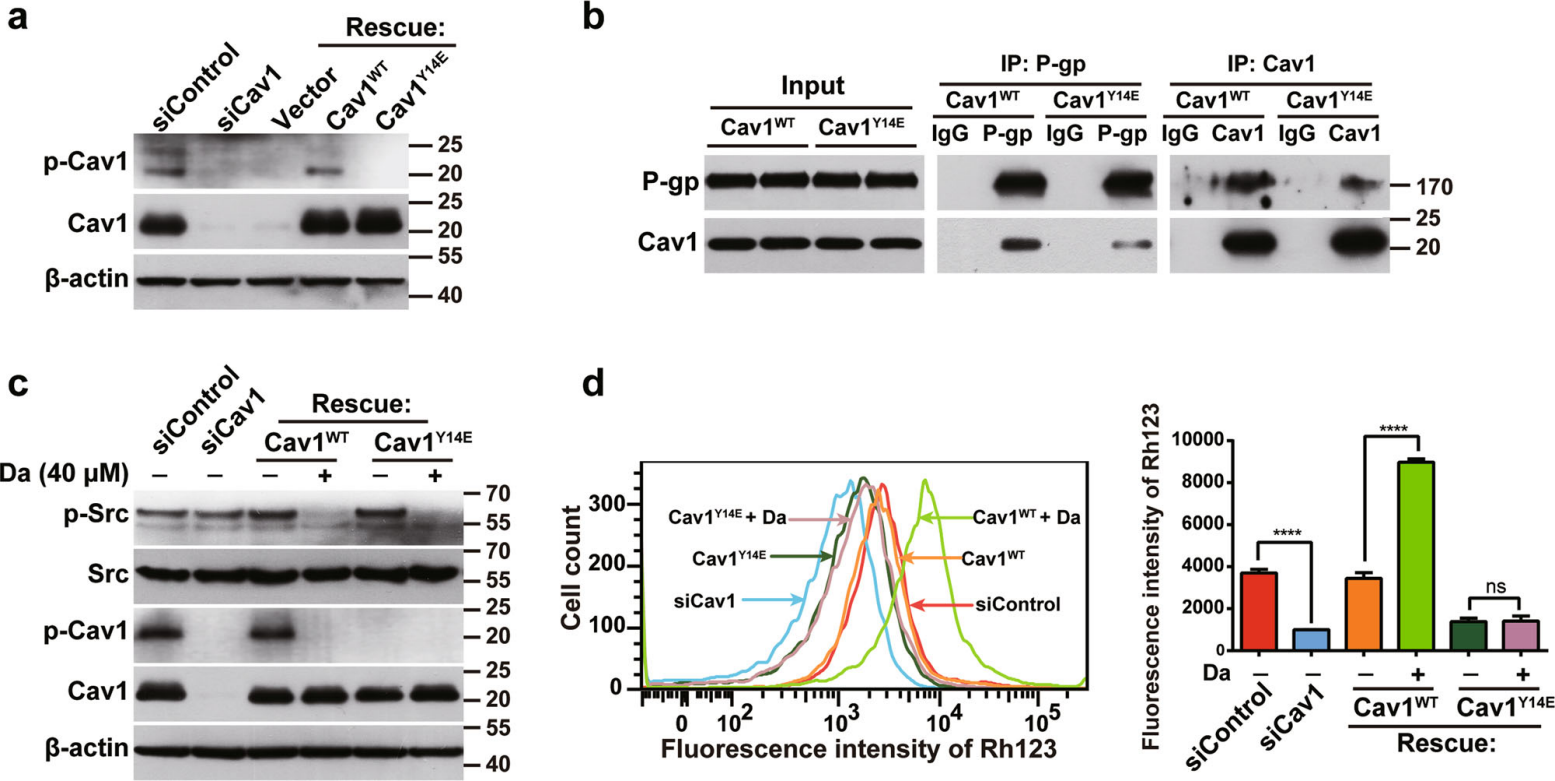
Phosphorylation of Cav1 decreases its binding to P-gp and enhances P-gp activity. **a** The expression of Cav1^WT^ and its mutants Cav1^Y14E^ were effectively rescued in Cav1-silenced MCF-7/ADR cells. Western blotting analysis of the expression level of Cav1 in cell lysates from control, Cav1-silenced cells, and Cav1^WT^- and Cav1^Y14E^-rescued cells. β-Actin was used as a loading control. **b** The phosphorylation of Cav1 decreases its binding to P-gp in drug-resistant breast cancer cells. The Cav1^WT^- and Cav1^Y14E^-rescued cells were lysed and immunoprecipitated with anti-P-gp or anti-Cav1 antibodies, and then analyzed by western blotting using anti-P-gp or anti-Cav1 antibodies. **c** Inhibition of Src kinase activity by using Dasatinib blocked the phosphorylation of Cav1 in Cav1^WT^-rescued cells. Western blotting analysis of the expression of total Cav1, phosphorylated Cav1, total Src, and phosphorylated Src in drug-resistant cancer cells treated with or without Dasatinib for 24 h; β-actin was used as the loading control. Note: the anti-phospho-Cav1 antibody does not recognize Cav1^Y14E^ mutant. **d** The phosphorylation of Cav1 by Src enhances the P-gp efflux activity. Rh123 efflux assay showed that Cav1 knockdown significantly enhances the efflux rate of Rh123 compared with control cells and rescued expression of Cav1^Y14E^ in Cav1-silenced cells significantly enhances the efflux rate of Rh123 compared with Cav1^WT^-rescued cells. Inhibition of Src activity in Cav1^WT^-rescued cells, but not in Cav1^Y14E^-rescued cells, increased intracellular Rh123 retention as measured by flow cytometry. The right panel shows the quantitative analysis of the Rh123 fluorescence intensity. Data were presented as mean ± SD, statistical analysis was performed using one-way ANOVA, ****P *<* 0.0001

To examine whether phosphorylation of Cav1 is necessary for its binding to P-gp, we silenced Cav1 expression by using an siRNA targeting its noncoding region. Then, we rescued Cav1 expression in Cav1-silenced cells with wild-type Cav1 (Cav1^WT^) or its phospho-mimicking mutant Cav1^Y14E^. As shown in Fig. 6a, Cav1 expression was effectively recovered in Cav1-silenced cells to levels similar to those in endogenous proteins. Then we investigated the binding ability of Cav1 to P-gp in Cav1-rescued cells through a Co-IP assay using anti-P-gp antibodies. Figure 6b showed that although anti-P-gp antibodies co-precipitated Cav1 in cell lysates from Cav1^WT^- and Cav1^Y14E^-rescued cells, the interaction of P-gp with Cav1 in the Cav1^Y14E^-expressing cells was notably attenuated compared with that in the Cav1^WT^-expressing cells. Consistently, a reciprocal Co-IP assay using anti-Cav1 antibodies also demonstrated that the binding ability of Cav1 to P-gp was decreased in Cav1^Y14E^-expressing cells compared with the Cav1^WT^-expressing cells. Therefore, these results indicated that phosphorylation of Cav1 attenuates its binding to P-gp. These data are also consistent with the above findings that knocking down the expression of Rack1 or Src inhibits Cav1 phosphorylation (Fig. 5a, d, e) and increases its interaction with P-gp (Fig. 4b, c, h). Next, we used Rh123 efflux assay to evaluate the function of P-gp in control, Cav1 knockdown, and Cav1-rescued cells. As shown in Fig. 6c, d, the accumulation of intracellular Rh123 dye was significantly decreased in Cav1 knockdown cells than in the control cells, indicating an increase of P-gp activity. In addition, re-expression of Cav1^Y14E^ showed enhanced P-gp efflux activity compared with that in Cav1^WT^-rescued cells, suggesting that the phosphorylated Cav1 abolishes its inhibitory effect on P-gp. Moreover, we treated Cav1-rescued cells with Src inhibitor and further performed Rh123 efflux assay. Interestingly, Cav1^WT^-expressing cells had a lower capacity to exclude Rh123 dye after Src inhibitor treatment, indicating that the P-gp pump function in Cav1^WT^-expressing cells was quenched by Src inhibitor, whereas Src inhibition had little effect on the efflux function of P-gp in Cav1^Y14E^-expressing cells. Therefore, these results suggest that the phosphorylation of Cav1 by Src positively regulates the P-gp pump activity in drug-resistant breast cancer cells.

### Rescued expression of Rack1 restores the chemoresistance

**Fig. 7:**
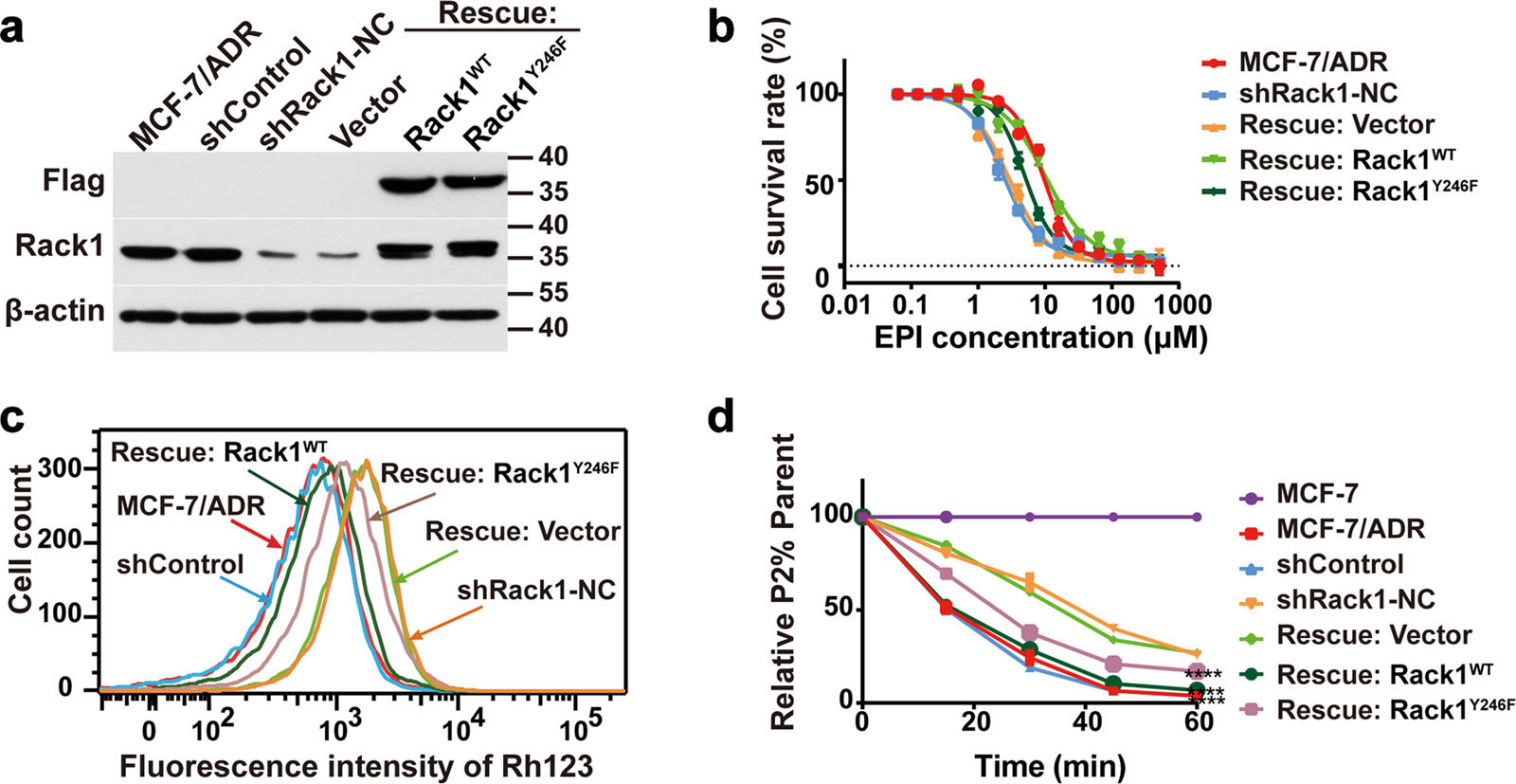
Rescued expression of Rack1 restores the chemoresistance. **a** Western blotting analysis of the expression level of Flag and Rack1 in cell lysates from wild-type, control, Rack1-silenced cells, and Rack1^WT^- and Rack1^Y246F^-rescued cells. β-Actin was used as a loading control. **b** Rescued expression of Rack1^WT^, but not Rack1^Y246F^ mutant, recovered cell viability in response to EPI. Wild-type, control, and Rack1-rescued cells were seeded in 96-well plates, then different concentrations of EPI were added into the cells, and drug sensitivity assay was performed by CCK8-based assay as described above. Values were expressed as mean ± SD from three independent experiment. **c** Rescued expression of Rack1^WT^ rather than Rack1^Y246F^ decreased the accumulation of Rh123 within cells as measured by flow cytometry. **d** Rescued expression of Rack1^WT^ significantly enhances the efflux rate of Rh123 compared with control or Rack1^Y246F^-rescued cells. Data were presented as mean ± SD, statistical analysis was performed using two-way ANOVA, ***P < 0.001, ****P < 0.0001 vs. control

Based on our findings that knockdown of Rack1 attenuated the activity of P-gp via destroying the P-gp/Rack1/Src complex in MDR cell lines and enhanced the sensitivity to EPI, we proposed that Rack1 restoration may regain chemoresistance in Rack1-silenced cells. As shown in Fig. 7a, b, stably silencing the expression of Rack1 decreased the cell survival rate in response to EPI, whereas rescuing with Rack1^WT^ significantly recovered cell viability (Supplementary Table 1). Meanwhile, re-expression of Rack1^Y246F^ mutant only partially recovered the cell viability. We next examined the efflux activity of P-gp by using flow cytometry. Figure 7c, d showed that rescuing with Rack1^WT^ decreased the accumulation of Rh123 within cells, whereas rescuing with Rack1^Y246F^ mutant faintly reduced the Rh123 accumulation in drug-resistant cells. Therefore, Rack1 is essential for P-gp efflux function and drug resistance.

## Discussion

MDR is the major cause for treatment failure in late stage of cancer^41^. The increased expression of ABC transporters is one of the most defined resistance mechanisms through which cancer cells efflux chemotherapeutic agents^42,43^. P-gp is the best-characterized pump to confer MDR. Thus, the blockage of P-gp activity is a very promising strategy to overcome MDR and effectively treat cancer^44,45^. However, the mechanism regulating P-gp activity remains poorly defined. Herein, we demonstrated that Rack1 modulates P-gp activity through mediating the interaction between P-gp and Src. Both Rack1 and Src confer resistance to EPI in breast cancer cells by modulating P-gp activity without affecting its protein level. Moreover, the binding of Rack1 to Src is required for Cav1 phosphorylation by Src, which increases the pump activity of P-gp and contributes to drug resistance. Hence, Rack1 interacts with Src and modulates the function of P-gp through regulating the Cav1 phosphorylation. Our findings also propose novel insights into the understanding of the molecular mechanism regulating the P-gp transporter activity.

The expression of Rack1 in cancer cells have been reported to be with cell proliferation^30^, migration^26^, invasion^46^, autophagy^47^, and apoptosis^48^. Recently, Rack1 overexpression is associated with targeted resistance in hepatocellular carcinoma and gastrointestinal stromal tumor^29,49,50^, and chemoresistance in gastric cancer and leukemia^51,52^. However, whether Rack1 is involved in P-gp-mediated drug resistance remains unclear. In this study, Rack1 knockdown in MDR cells increased drug susceptibility without affecting the P-gp protein level. Nevertheless, Rack1-silenced cells trap more cytotoxic drugs intracellularly, indicating the decrease of the P-gp activity. This finding is in accordance with the slowdown of Rh123 dye efflux rate in Rack1-knockdown cells. Together, these findings suggest that Rack1 regulates drug resistance through modulating the efflux pump activity of P-gp, but not its protein level. Rack1 and Src are novel binding proteins of P-gp in drug-resistant cells^39^ and Src is also the binding partner and the upstream kinase of Rack1^38^. The elevated expression of Src is also associated with drug resistance in many cancer types^53^. Therefore, it is reasonable to suppose that Src is also associated with P-gp-mediated drug resistance. As expected, Src knockdown significantly decreased cell viability in resistant cells in response to EPI without altering the protein level of P-gp. Moreover, Src-silenced cells had a lower capacity to exclude its substrates outside the cells, indicating the reduction in the pump activity of P-gp. The blockage of Src kinase activity by inhibitors makes cancer cells sensitive to EPI, whereas the expression of P-gp was not altered. Similarly, the P-gp activity was also impaired in Src inhibitor-treated cells. These data further confirmed the requirement of Src kinase in the regulation of P-gp pump activity in resistant cells. Collectively, both Rack1 and Src bind to P-gp and regulate its transport function.

The mechanism whereby Rack1 and Src modulate P-gp activity is very worthy of investigation. It has been established that the transport function of P-gp is modulated by Cav1. The increased binding of Cav1 to P-gp inhibits its transport activity, whereas the attenuated interaction between Cav1 and P-gp enhances its pump function. Herein, we demonstrated that the binding of Cav1 to P-gp in Rack1- or Src-silenced cells is considerably increased compared with that in the control cells. This finding is in accordance with the observations that silencing the expression of Src or Rack1 enhances drug sensitivity by suppressing P-gp activity. Hence, Rack1 and Src modulate P-gp activity through regulating the binding of Cav1 to P-gp in drug-resistant cells. Tyrosine phosphorylation of Cav1 also regulates P-gp activity. In this study, either knocking down the expression of Src by siRNAs or inhibiting its kinase activity by using inhibitors can significantly block Cav1 phosphorylation. Given that Rack1 binds to Src and regulates the transport function of P-gp, we speculated that Rack1 may be involved in Cav1 phosphorylation. Notably, knockdown of Rack1 apparently abrogated the phosphorylation of Cav1. Moreover, the re-expression of Rack1 in the Rack1-silenced cells rescued the phosphorylation of Cav1, further confirming that Rack1 is required for Cav1 phosphorylation. Furthermore, the rescued expression of Rack1 also recovered the transport function of P-gp and enhanced resistance to EPI in vitro. Altogether, Rack1 and Src modulate P-gp activity by regulating Cav1 phosphorylation.

We have already established that P-gp forms a protein complex with Rack1 and Src in drug-resistant cells, and Rack1 is essential to maintain the integrity of this protein complex^39^. Herein, we showed that P-gp is immunoprecipitated with Src, Rack1, and Cav1 under endogenous condition. Src also interacted with P-gp, Rack1, and Cav1; these data raised a possibility that Rack1 may recruit these proteins together to facilitate the phosphorylation of Cav1 by Src. However, anti-Rack1 antibodies could only coprecipitate with P-gp and Src but not with Cav1. Likewise, Cav1 only interacted with P-gp and Src, but failed to coprecipitate with Rack1. These results indicate that the regulatory effect of Rack1 on Cav1 phosphorylation is not obtained through the direct interaction between these two proteins. A possible explanation is that both Cav1 and Rack1 can bind P-gp, although there is no directly binding between these two proteins. Since Rack1 is required for the binding of Src to P-gp, this binding provides an accessible interaction between Src and Cav1, facilitating the phosphorylation of Cav1 by Src. In addition, we further demonstrated that the interaction of P-gp with Cav1 in the phospho-mimicking Cav1^Y14E^-expressing cells was notably attenuated compared with that in the Cav1^WT^-expressing cells, suggesting that the attenuation of the binding of Cav1 to P-gp enhances P-gp activity. These findings indicated that the phosphorylation of Cav1 by Src abrogates its inhibitory effect on P-gp. As expected, re-expression of Cav1^Y14E^ showed enhanced P-gp efflux activity compared with that in Cav1^WT^-rescued cells. Moreover, the P-gp efflux activity in Cav1^WT^-expressing cells can be quenched by Src inhibitor, whereas Src inhibition has little effect on the pump function of P-gp in Cav1^Y14E^-expressing cells. Therefore, these results suggest that the phosphorylation of Cav1 by Src positively regulates the P-gp pump activity in drug-resistant breast cancer cells. Collectively, these results support a novel role for Rack1 as a key modulator in drug resistance through its interaction with Src and regulation of Cav1 phosphorylation, and thereby eliminating the inhibitory effect of Cav1 on P-gp.

In summary, Rack1 and Src confer drug resistance by modulating the transport function of P-gp without altering its protein level. Rack1 and Src regulate P-gp activity by modulating the Cav1 phosphorylation. Furthermore, Rack1 functions as a signaling hub by mediating the interaction between Src and P-gp, and thereby facilitating the phosphorylation of Cav1 by Src. The phosphorylated Cav1 abolishes its inhibitory effect on P-gp. This discovery extends our knowledge of the activation mechanisms regulating P-gp transporter activity.

## Materials and methods

### Cell lines and reagents

Human breast cancer cell lines SK-BR-3 were obtained from the American Type Culture Collection (ATCC; Manassas, Virginia, USA). The MDR variant SK-BR-3/EPR was established in our previous study by means of a stepwise long-term exposure to an increasing concentration of EPI^54^. The human breast cancer cell line MCF-7 and their drug-resistant variants MCF-7/ADR were obtained from Henry Ford Hospital (Detroit, MI, USA). The cells were maintained in a modified RPMI medium (Hyclone, Logan, Utah 84321, USA) supplied with 10% fetal bovine serum (FBS, Gibco, Australia) at 37 °C, 5% CO_2_ conditions. MCF-7/ADR and SK-BR-3/EPR cell lines were cultured in complete growth medium supplied with 0.5 μM EPI (Hisun Pfizer Pharmaceuticals, Zhejiang, China) to maintain their drug-resistant property. HEK-293T cells (ATCC) were maintained in Dulbecco’s modified Eagle’s medium/high glucose (Hyclone, Logan, Utah 84321, USA) supplied with 10% FBS at 37 °C under 5% CO_2_ conditions. Drug-resistant cell lines were cultured in drug-free medium at least 1 week before conducting experiments. All stable transduced cell lines in this study were cultured in the corresponding complete growth medium supplied with 50 μg/mL of hygromycin B (Solarbio Science & Technology Co., Beijing, China). All cell lines used in this study were regularly authenticated by morphological observation and tested for the absence of mycoplasma contamination.

### siRNA, vector construction, lentivirus packing and infection, and stable cell line selection

Three Rack1-specific stealth siRNAs, Src-specific stealth siRNAs, and a negative control siRNA were purchased from Invitrogen (Life Technologies, Carlsbad, CA, USA). Cav1-specific siRNA targeting its noncoding region was obtained from Genepharma (Shanghai GenePharma Co., Ltd). The sequences of the siRNAs are shown in Table 1. Transient transfection was performed as described previously^39^. Rack1-specific shRNA sequence targeting its noncoding region (shRack1-NC: 5′-TGGCACACGCTAGAAGTTTATGG-3′) was subcloned into a lentiviral vector, pLko.1-hygromycin, in the BamH I and Age I cloning sites. Full-length wild-type Rack1 (Rack1 WT) was amplified by PCR with the following primers: upper: 5ʹ-GAGAGCTAGCATGACTGAGCAGATGACCCT-3ʹ, lower: 5ʹ-GAGAGCGGCCGCCTACTTGTCGTCATCGTCTTTGTAGTCGCGTGTGCCAATGG-3ʹ, and cloned into a lentiviral vector pCDH-hygromycin in the Nhe I and Not I cloning sites. Then, this vector was used as a template to create a Rack1 mutant through site-directed mutagenesis. The mutation of TAC (Y) to TTC (F) at the amino acid 246-encoding position that generated Rack1 Y246F mutant and where tyrosine 246 was replaced by phenylalanine. Full-length wild-type Cav1 (Cav1 WT) was amplified by PCR with the following primers: upper: 5ʹ-GAGAGAATTCATGTCTGGGGGCAAATACGTAG-3ʹ, lower: 5ʹ- GAGAGGATCCATTGTTGCTGTATTAGCAACTTGGAAC-3ʹ, and cloned into the lentiviral vector pCDH-hygromycin in the EcoR I and BamH I cloning sites. The mutation of TAC (Y) to GAG (E) at the amino acid 14-encoding position generated a Cav1 (Y14E) phosphor-tyrosine mimicking mutant and where tyrosine 14 was replaced by glutamic acid. All the constructs were further confirmed by double-enzyme digestion and DNA sequencing. Lentivirus production was achieved by a standard three-plasmid packaging system. In brief, HEK-293T cells were seeded in 10 cm dish at 60% confluence 12 h before transfection. Then, the lentiviral vector and two packaging plasmids were co-transfected into HEK-293T cells by using polyethyleimine. After transfection for 48 h, the virus-containing supernatants were collected and used to infect the MCF-7/ADR and SK-BR-3/EPR cells. Stable cell lines were selected and maintained by using 50 μg/mL of hygromycin B.

**Table 1:** siRNA target sequences used in this study

Name	Sequence
siRack1-1#	Upper: 5′-UAUCUCGAGAUCCAGAGACAAUCUG-3′
	Lower: 5′-CAGAUUGUCUCUGGAUCUCGAGAUA-3′
siRack1-2#	Upper: 5′-ACGAUGAUAGGGUUGCUGCUGUUGG-3′
	Lower: 5′-CCAACAGCAGCAACCCUAUCAUCGU-3′
siRack1-3#	Upper: 5′-AUAACCACAUCACUAACAAAGUGGG-3′
	Lower: 5′-CCCACUUUGUUAGUGAUGUGGUUAU-3′
siSrc-1#	Upper: 5′-CAGCAGCUGGUGGCCUACUACUCCA-3′
	Lower: 5′-UGGAGUAGUAGGCCACCAGCUGCUG-3′
siSrc-2#	Upper: 5′-GAGCCCAAGCUGUUCGGAGGCUUCA-3′
	Lower: 5′-UGAAGCCUCCGAACAGCUUGGGCUC-3′
siSrc-3#	Upper: 5′-CACCAGACGGGAGUCAGAGCGGUUA-3′
	Lower: 5′-UAACCGCUCUGACUCCCGUCUGGUG-3′
siCav1	Upper: 5′-GCUUUGUGAUUCAAUCUGUAA-3′
	Lower: 5′-UUACAGAUUGAAUCACAAAGC-3′

### Western blotting assay

Western blotting assay was performed as described previously^55^. Briefly, the cells were lysed with 1 × SDS lysis buffer and the cell lysates were quantified by using NANODROP 2000. Approximately 40 μg of protein were separated by SDS-polyacrylamide gel electrophoresis (PAGE) and transferred to polyvinylidene difluoride membranes. The membranes were blocked by 5% defatted milk and incubated with primary antibodies overnight at 4 °C. The following antibodies were used: P-gp (1:1000, Santa Cruz Biotechnology, Santa Cruz, CA), Src (1:1000, Cell Signaling Technology, Beverly, MA, USA), p-Src (1:1000, Cell Signaling Technology, Beverly, MA, USA), Rack1 (1:1000, Santa Cruz Biotechnology, Santa Cruz, CA), Cav1 (1:5000, Abcam), p-Cav1 (1:1000, Abcam), Flag (1:1000, Sigma, St. Louis, MO, USA), and β-actin (1:10000, Sigma-Aldrich). After washing thrice with 1 × TBST, the membrane was incubated with the corresponding horseradish peroxidase-linked secondary antibodies (BIO-RAD, Inc., USA), followed by detection using an ECL kit (Millipore, Billerica, MA, USA) according to the manufacturer’s protocol. β-Actin was used as a loading control.

### Co-IP analysis

Co-IP analysis was performed as described previously^55^. Briefly, cells grown in 10 cm dishes, washed with ice-cold phosphate-buffered saline (PBS), and lysed with Tris-Triton X-100-based cell lysis buffer on ice. The cell lysates were collected and centrifuged at 12,000 × *g* for 15 min at 4 °C. The supernatant was transferred to a new tube and precleared with protein G-conjugated agarose beads. Then, 1 μg of each corresponding antibody (P-gp, Src, Rack1, or Cav1) was added into the supernatant and further incubated overnight at 4 °C for the enrichment of the antigen–antibody complex. The immunocomplex was precipitated with protein G-agarose beads. The beads were then washed with cell lysis buffer and boiled with 1 × SDS buffer at 95 °C for 10 min. Next, the bound proteins were separated by SDS-PAGE, followed by western blotting analysis.

### Rh123 efflux assay

Rh123 efflux assay was performed as described previously with minor modification^56^. In brief, cells at the logarithmic phase were collected with trypsin, washed with PBS, and resuspended in cell culture medium containing 1.0 μg/mL of Rh123 dye at a density of 1 × 10^6^ cells/mL. The cell suspension was incubated for 30 min at 37 °C and 5% CO_2_ to allow the uptake of Rh123. Then, the cells were centrifuged, washed three times with PBS, and incubated in Rh123-free medium at 37 °C for 0, 15, 30, 45, and 60 min. At each time point, the cells were washed twice with PBS, resuspended with 200 µL PBS, and immediately detected by flow cytometry by using the excitation and emission wavelengths at 488 and 530 nm, respectively. The Rh123 dye-positive cell counts and the mean fluorescence intensity were used for the evaluation of the efflux pump function of P-gp. The assays were performed in triplicate.

### IC_50_ assay

IC_50_ assay was performed using a CCK8 assay as described previously^39^. In brief, cells were seeded into a 96-well plate at a density of 5.0 × 10^3^ cells per well and incubated for 24 h. Then, EPI was diluted with fresh medium at a gradient concentration of 0, 0.125, 0.25, 0.5, 1, 2, 4, 8, 16, 32, 64, and 128 μM, and added into the cells. After incubation for 72 h, the medium was replaced with 100 μL of fresh medium containing 10% CCK8 reagent and the cells were further cultured for 3 h. Cell viability was determined by measuring the absorbance at 450 nm on a micro-ELISA reader. The assay was performed in triplicates for each EPI concentration and repeated thrice. The IC_50_ value was calculated by GraphPad Prism 6.0 software (GraphPad Software, La Jolla, CA, USA).

### Immunofluorescence assay

Cells were seeded at 3 × 10^4^ cells/well in a 12-well plate containing glass coverslip and cultured for 24 h. Control and Rack1-silenced cells were initially incubated with 2 μM of EPI for 2 h, then the cells were incubated with EPI-free medium for additional 1 h. Afterward, the cells were fixed with 4% paraformaldehyde, permeabilized with 0.1% Triton X-100, counterstained with 1.0 ng/mL of DAPI (4′,6-diamidino-2-phenylindole) for nuclei. The coverslips were mounted with Mowoil-based anti-quenching medium and imaged by fluorescence microscope (EVOS, Life Technologies, Carlsbad, CA, USA).

### Statistical analysis

All the data were presented as mean ± SD and repeated in three independent trials. The differences between the two groups were compared by two-tailed Student’s *t*-test. For multiple group comparison, two-way analysis of variance was performed. All data were analyzed with GraphPad Prism 6.0 software and *P* < 0.05 was considered statistically significant.

## Supplementary information

supplementary tables

Supplementary Figure 1
