# Supplementary material for: Rack1 mediates Src binding to drug transporter P-glycoprotein and modulates its activity through regulating Caveolin-1 phosphorylation in breast cancer cells
Source: Cell Death Dis. 2019 May 21;10(6):394. doi: 10.1038/s41419-019-1633-y (PMC6529477; doi:10.1038/s41419-019-1633-y)
Supplement: Supplementary file 1 — supplementary tables [file 41419_2019_1633_MOESM1_ESM.docx]

**Supplementary Tables**

**Table 1.** IC_50_ of EPI in MCF-7/ADR cells transfected with control, Rack1 or Src specific siRNAs, and in Rack1-silenced cells rescued with control, Rack1^WT^ or Rack1^Y246F^ mutant.

| Cell type | IC_50_ (μM) | *P-Value* |
| --- | --- | --- |
| MCF-7/ADR  siControl  siRack1-1#  siRack1-2#  siSrc-1#  siSrc-2#  siSrc-3#  shRack1-NC  Rescue: pCDH  Rescue: Rack1^WT^  Rescue: Rack1^Y246F^ | 12.99 ± 0.844  12.90 ± 1.400  5.47 ± 0.845  4.01 ± 0.541  5.14 ± 1.042  3.29 ± 0.078  5.42 ± 2.333  4.03 ± 1.323  3.78 ±1.736  12.23 ± 3.100  7.84 ± 3.431 | ns  -  <0.0001  <0.0001  <0.0001  <0.0001  <0.0001  <0.0001  -  P<0.001  ns |

**Table 2.** IC_50_ of EPI in SK-BR-3/EPR cells transfected with control, Rack1 or Src specific siRNAs.

| Cell type | IC_50_ (μM) | *P-Value* |
| --- | --- | --- |
| SK-BR-3/EPR  siControl  siRack1-1#  siRack1-2#  siSrc-1#  siSrc-2#  siSrc-3# | 16.11 ± 0.882  16.17 ± 0.790  5.67 ± 0.007  4.47 ± 0.003  2.52 ± 0.555  5.06 ± 0.449  3.79 ± 0.565 | ns  -  <0.0001  <0.0001  <0.0001  <0.0001  <0.0001 |

**Table 3.** IC_50_ of EPI in MCF-7/ADR cells treated with DMSO, different concentration of dasatinib or saracatinib.

| Inhibitor | IC_50_ (μM) | *P-Value* |
| --- | --- | --- |
| DMSO  Sa 10 μΜ  Sa 20 μM  Da 60 μΜ  Da 80 μΜ | 12.22 ± 1.709  1.27 ± 0.839  0.50 ± 0.199  4.02 ± 0.457  2.17 ± 2.333 | -  <0.0001  <0.0001  <0.0001  <0.001 |

**Table 4.** IC_50_ of EPI in SK-BR-3/EPR cells treated with DMSO, different concentrations of dasatinib or saracatinib.

| Inhibitor | IC_50_ (μM) | *P-Value* |
| --- | --- | --- |
| DMSO  Sa 10 μΜ  Sa 20 μM  Da 60 μΜ  Da 80 μΜ | 16.17 ± 0.882  1.48 ± 0.698  0.37 ± 0.003  1.12 ± 0.035  0.63 ± 0.023 | -  <0.0001  <0.0001  <0.0001  <0.0001 |
