# Supplementary material for: Rack1 mediates Src binding to drug transporter P-glycoprotein and modulates its activity through regulating Caveolin-1 phosphorylation in breast cancer cells
Source: Cell Death Dis. 2019 May 21;10(6):394. doi: 10.1038/s41419-019-1633-y (PMC6529477; doi:10.1038/s41419-019-1633-y)
Supplement: Supplementary file 2 — Supplementary Figure 1 [file 41419_2019_1633_MOESM2_ESM.docx]

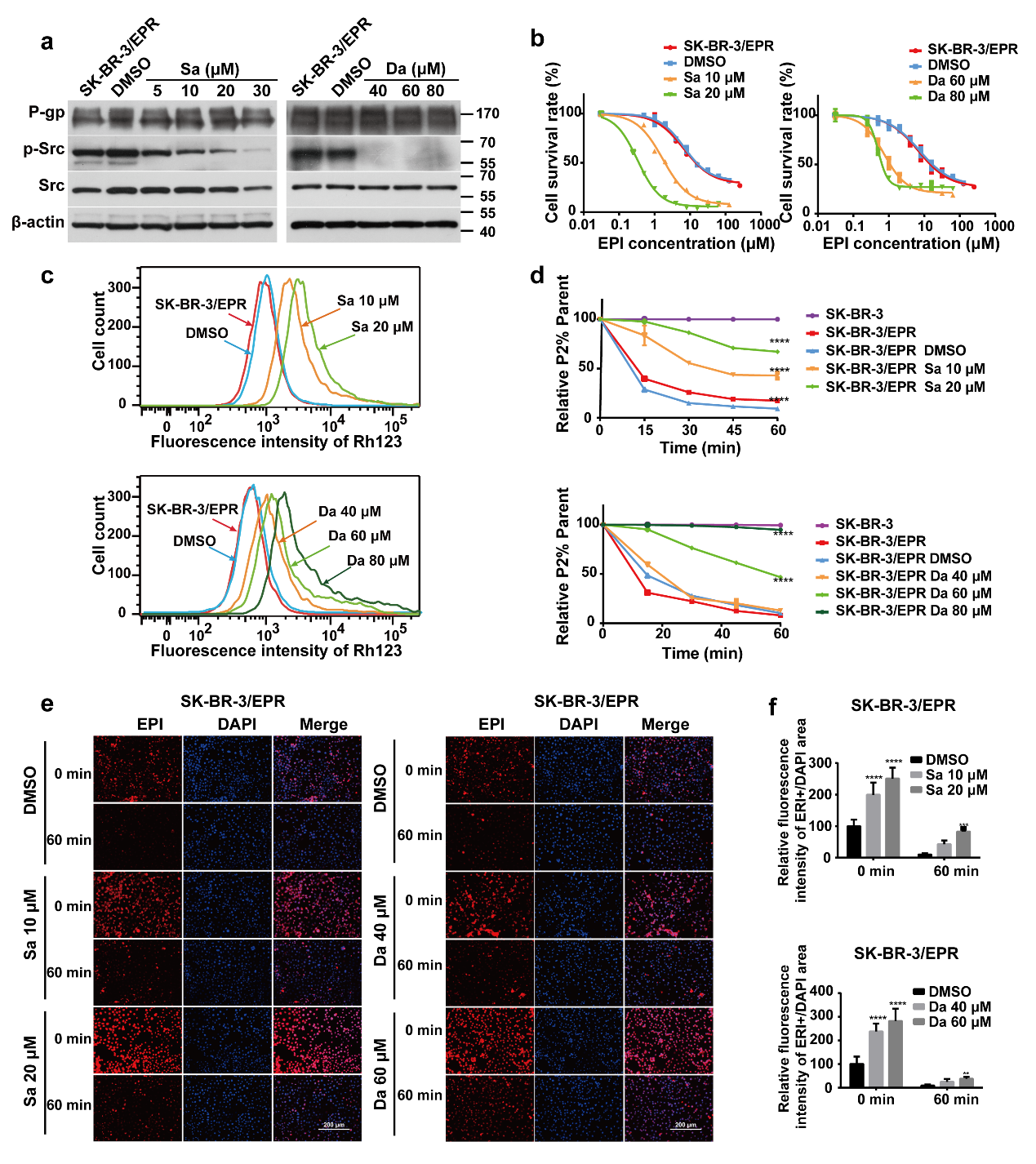


**Supplementary Fig 1. Inhibition of Src kinase activity in SK-BR-3/EPR cells increased chemosensitivity in MDR cells.** (a) Western blotting analysis of the expression of P-gp, total Src and phosphorylated Src in SK-BR-3/EPR cells after Saracatinib or Dasatinib treatment for 24 h; (b) Treatment with Src inhibitors in MDR cells significantly enhanced drug sensitivity to EPI in comparison with control cells. The cells were pretreated with Src inhibitors for 24h, then different concentrations of EPI were added into the cells, and drug sensitivity assay was performed by CCK8-based assay as described above. Values were expressed as mean ± SD from three independent experiment; (c) Inhibition of Src activity by two inhibitors in drug resistant cells enhanced intracellular Rh123 retention in comparison with wild type and control cells as measured by flow cytometry; (d) Src inhibitors treatment significantly decreases the efflux rate of Rh123 compared with control cells. Data was presented as mean ± SD, statistical analysis was performed using two-way ANOVA, ****P* < 0.001, *****P* < 0.0001 versus solvent control; (e) Src inhibitors treatment significantly increased cellular EPI retention compared with control cells. (f) Quantification of EPI fluorescence intensity in Fig 3e by using Image J (NIH, Bethesda MD, UAS) software. ****P*<0.001 versus DMSO control, data are resented as mean ± SD, statistical analysis was calculated by two-way ANOVA.
